# Targeting Lung Cancer Cell Motility Using Microbeam Radiation Therapy

**DOI:** 10.3390/cells15020107

**Published:** 2026-01-07

**Authors:** Ömer Dağkazanlı, Aleksandra Čolić, Rainer Lindner, Stefan Bartzsch, Stephanie E. Combs, Thomas E. Schmid, Marina Santiago Franco

**Affiliations:** 1 Department of Radiation Oncology, TUM School of Medicine and Health and Klinikum Rechts der Isar, University Hospital of the Technical University of Munich, Ismaninger Straße 22, 81675 Munich, Germany; omer.dagkazanl@tum.de (Ö.D.); aleksandra.colic@tum.de (A.Č.); stefan.bartzsch@tum.de (S.B.); stephanie.combs@tum.de (S.E.C.); 2 Institute of Radiation Medicine (IRM), Helmholtz Zentrum München GmbH, German Research Center for Environmental Health, 85764 Neuherberg, Germany; rainer.lindner@helmholtz-munich.de

**Keywords:** invasion, irradiation, metastasis, migration, *NF-κB*, CD44, spatial fractionation

## Abstract

Radiotherapy (RT) is currently among the standard treatments for lung cancer. However, in vitro studies have revealed that irradiation can increase lung cancer cell motility. This way, RT could potentially enhance the malignancy of solid tumors post-treatment, promoting metastasis. Therefore, there is a continued need to continue evolving RT modalities into safer and more effective treatments. The present study compares the impact of the broad beam (BB) and the spatially fractionated modality of microbeam radiation therapy (MRT) on the motility of A549 lung cancer cells. Our data corroborates previous findings that showed BB irradiation is a promoter of cell motility. For MRT, however, we observed a prevention of cellular migration. A significant reduction in *NF-κB* expression was observed only when A549 cells were irradiated with MRT, indicating a potential mechanism behind these findings. Finally, our data supports potential issues regarding MRT irradiation of key components of the tumor microenvironment, such as fibroblasts. Co-culturing A549 cells with MRT-irradiated MRC-5 lung fibroblasts led to increased tumor cell invasion, not observed when the fibroblasts received BB irradiation.

## 1. Introduction

Lung cancer (LC) is not only the cancer with the highest incidence worldwide but also the leading cause of death by cancer. The latest global cancer statistics by world regions (2022) reported close to 20 million new cancer cases and around 9.7 million deaths by cancer. Of those, LC consisted of 2.48 million new cases (12.4%) and 1.82 million deaths (18.7%) [1]. The poor prognosis can be attributed to the usual late detection of LC, posing challenges to the treatment [2]. Therefore, improvements in tumor detection and therapy modalities are urgently required. Currently, the standard treatments for lung cancer include radiotherapy (RT), chemotherapy, and surgery [3]. Different modalities of RT, such as stereotactic body radiotherapy, intensity-modulated radiotherapy, and particle beam therapy (carbon-ion and proton beam) are currently used for LC management [4]. This has allowed for a reduction in the incidence of radiation pneumonitis and radiation-induced pulmonary fibrosis, dose-limiting and potentially fatal complications of RT to the lungs. However, radiation-induced lung injuries remain a substantial clinical issue [5]. Contributing to the sometimes unsatisfactory outcomes of RT for LC could be its potential to enhance the malignancy of post-treatment remaining tumor cells by increasing their motility. Ineffective RT of solid tumors is suggested to enhance metastasis formation, as the tumor and its microenvironment are modified phenotypically and genetically by the treatment. Literature evidence shows that cells surviving RT metastasize more frequently in vivo, which could potentially lead to recurrence in patients [6]. This phenomenon of increased tumor cell motility post RT is extensively studied in glioblastoma models both in vitro and in vivo. Different molecular mechanisms responsible for this effect have been reported and were recently reviewed by Franco and coworkers (2024) [7]. Radiation has also been reported to increase motility of LC cells in different in vitro studies [8,9,10,11,12]. Taken together, the need for safer and more effective treatment highlights the need to continue evolving RT modalities. Microbeam radiation therapy (MRT) is a novel pre-clinical modality that was first explored by Daniel Slatkin and coworkers in the 1990s [13]. MRT is a form of spatially fractionated radiation therapy, where the microbeam field consists of micrometer-sized planar beams separated by center-to-center (ctc) distances of a few hundred micrometers. This leads to high-dose regions (“peaks”) and low-dose regions (“valleys”) in the target. This modality has proven to be better tolerated by a range of normal tissues, maintaining or even increasing tumor control [14,15]. Despite being technically challenging, Schültke et al. (2021) proved that the delivery of MRT to the lungs is feasible in a mouse model [16]. Since then, efforts are ongoing in order to confirm if MRT can increase the therapeutic window in LC [17]. Although MRT is still a pre-clinical modality, a compact X-Ray tube that will allow its translation to the clinical setting is under active development [18]. To the best of our knowledge, there has been no report on how MRT impacts tumor cell motility as compared to conventional irradiation. In the present study, we investigated how BB and MRT irradiation affects A549 LC cell line motility.

## 2. Materials and Methods

### 2.1. Cell Culture and Cell Irradiation

The A549 cell line (human lung adenocarcinoma; ATCC CCL-185™) was cultured in Dulbecco’s modified Eagle medium (DMEM) high glucose (Sigma, Taufkirchen, Germany). The normal tissue MRC-5 cell line (human lung fibroblasts; ATCC CCL-171™) was cultured in DMEM/F12 (Gibco, Waltham, MA, USA). Broad beam irradiation was performed at the RS225 (X-Strahl, Brownhills, UK) with 220 kVp photons filtered with 0.15 mm of copper, with a dose rate of 0.89 Gy/min. MRT irradiation took place at a XenX irradiator (X-Strahl, Brownhills, UK), with a setup previously developed by our research group [19,20]. A custom-made tungsten microbeam multislit collimator with slit widths of 50 µm separated by center-to-center (ctc) distances of 400 µm was used to shape the MRT field. MRT irradiation was performed using a 225 kVp photon spectrum filtered with 1 mm aluminum, followed by additional 2 mm aluminum and 2 mm polymethylmethacrylate (PMMA) directly above the MRT collimator. The peak dose rate was 4.95 Gy/min and the valley dose rate 0.24 Gy/min, meaning a peak-to-valley dose ratio (PVDR) of 20.6. Irradiation doses were 0, 4, and 6 Gy for BB, and the equivalent uniform dose (EUD) was applied for MRT. The EUD values were calculated based on linear quadratic fit parameters for each cell line previously reported by our research group: A549: α = 0.419 Gy^−1^, β = 0.018 Gy, α/β = 23.2 Gy and MCR-5: α = 0.637 Gy^−1^, β = 0.0458 Gy, α/β = 13.9 Gy [21].

### 2.2. LDH Cytotoxicity Assay

The cytotoxicity of the different irradiation doses applied to the different cell lines was verified with an LDH assay (Enzo, Farmingdale, NY, USA). Initially, 9 × 10^4^ A549 or MRC-5 cells in 0.5 mL of 2% fetal calf serum (FCS) medium were seeded in 24-well plates and allowed to attach overnight at 37 °C and 5% CO_2_ in the incubator. Before irradiation, medium was exchanged for 0.5 mL of 10% FCS, and cells were treated with sham or different irradiation doses of BB (4, 6 Gy) or MRT (EUD to 4 and 6 Gy). Cells were incubated for 72 h and then the released LDH was quantified in the supernatant according to the manufacturer’s instructions. Briefly, 100 μL of supernatant was transferred to a well on a 96-well plate followed by 100 μL of the Working Solution. The plate was incubated at room temperature for 30 min in the dark. Then, 50 μL of the Stop Solution was added to each well, and the absorbance at 490 nm was immediately measured on the Infinite M200 microplate reader (Tecan, Männedorf, Switzerland). As a cytotoxicity positive control, cells were completely lysed by adding 50 μL of lysis buffer to wells containing untreated cells and incubated for 30 min. Medium only was used as a background control.

### 2.3. Microscopy Analysis

For microscopy, 1.5 × 10^4^ A549 or 2 × 10^4^ MRC-5 cells in 0.3 mL of 2% FCS medium were seeded in 8-well slides (Ibidi, Gräfelfing, Germany) and allowed to attach overnight at 37 °C and 5% CO_2_ in the incubator. Before irradiation, medium was exchanged for 0.3 mL of 10% FCS, and cells were treated with sham or different irradiation doses of BB (4, 6 Gy) or MRT (EUD to 4 and 6 Gy). For conditioned medium (CM) experiments, instead of irradiation, A549 cells received MRC-5 CM at a proportion of 1:3. Cells were incubated for 72 h and then fixed with methanol and acetic acid (3:1).

#### 2.3.1. Evaluation of Cell Morphology

For cell morphology evaluation on a bright field, fixed cells were stained with crystal violet for 5 min and then rinsed with phosphate-buffered saline (PBS). Images were acquired on the BZ-9000 microscope (Keyence, Frankfurt am Main, Germany). Cell area measurements were performed using Fiji ImageJ software (version 1.54g) according to the protocol described by Nurmagambetova et al. (2023) [22].

#### 2.3.2. Immunofluorescence Microscopy

For immunofluorescence microscopy, fixed cells were blocked for 45 min with a solution of 5% FCS in PBS followed by incubation with conjugated antibody (CD44, E-cad) for 3 h at 37 °C and 5% CO_2_. For non-conjugated antibodies (ɣH2Ax), samples were incubated with primary antibody for 2 h and secondary (Alexa Fluor^®^ 488) for 1 h at 37 °C and 5% CO_2_. Cells were then rinsed with PBS and incubated for 10 min with a Hoechst 33342 solution (Thermo Fisher Scientific, Waltham, MA, USA). Fluorescence microscopy images were acquired on a Zeiss Axio Observer (Zeiss, Oberkochen, Germany) microscope. CD44 (Alexa Fluor^®^ 647 anti-human CD44 Antibody) and E-cadherin (Alexa Fluor^®^ 647 anti-mouse/human CD324 Antibody) were obtained from Biolegend, San Diego, CA, USA. Primary ɣH2Ax (Anti-phospho-Histone H2A.X (Ser139) Antibody, clone JBW301) antibody was obtained from Merck, Darmstadt, Germany. Secondary Alexa Fluor^®^ 488 (Goat anti-Mouse IgG (H + L) Cross-Adsorbed Secondary Antibody) was obtained from Thermo Fisher Scientific, USA.

### 2.4. Cell Adhesion Assay

A cell adhesion assay was performed as previously described by Jung et al. (2007) with minor modifications [8]. Briefly, cell culture flasks containing A549 cells at a confluency of around 70% were incubated with DMEM supplemented with 2% FCS overnight. On the next day, cells were harvested by adding 5 mL of 1 mM EDTA and incubating at 37 °C for 10 min. Detached cells were counted, and 2 × 10^5^ cells in 2 mL were seeded in non-adherent 24-well plates. Cells in suspension were treated with sham or different irradiation doses of BB (4, 6 Gy) or MRT (EUD to 4 and 6 Gy). Following irradiation, cells were immediately seeded on 96-well plates (2 × 10^4^ cells/well) and incubated for 30 min. Non-adherent cells were then removed after this incubation period by washing twice with PBS. Attached cells were quantified by incubating cells with Alamar Blue (Thermo Fisher Scientific, Waltham, MA, USA) for 4 h at 37 °C. Absorbance levels were measured using the Infinite M200 microplate reader (Tecan, Männerdorf, Switzerland) at 570 nm and 600 nm (reference) wavelengths.

### 2.5. Two-Dimensional Migration Assay

Two-well culture inserts in a 35 mm µ-dish (Ibidi, Gräfelfing, Germany) were used for a 2D migration assay. These inserts create a cell-free gap, which allows for uniform, highly reproducible quantitative migration experiments. Exponentially growing A549 cells were seeded at 2.1 × 10^4^ cells per insert well in 2% FCS medium. A low FCS concentration was used to suppress proliferation, which can be a confusing factor in migration experiments. Dishes were stored in the incubator overnight at 37 °C and 5% CO_2_. The following day, cells were treated with sham or different irradiation doses of BB (4, 6 Gy) or MRT (EUD to 4 and 6 Gy). Right after irradiation, inserts were carefully removed, cells were washed with PBS, and medium containing 2% FCS was replaced. At least two images (5× magnification) along the cell-free area were taken right after gap formation, marking the 0 h timepoint (Primovert microscope, Zeiss, Oberkochen, Germany). Dishes were stored in the incubator at 37 °C and 5% CO_2_ for 24 h, allowing for cell migration. Afterwards, images were again taken as stated before. The contrast-phase images were analyzed using the open-source image processing software ImageJ (version 1.54g) with a plugin the recognizes gap areas automatically (wound_healing_size_tool.ijm) [23]. The percentage of gap closure was calculated as follows:

(1) $$Gap\;closure\;\left(\%\right)=\left(\frac{A\;0\;h-A\;24\;h}{A\;0\;h}\right)\;\times \;100\%$$

where ‘A 0 h’ is the cell-free area at the 0 h timepoint and ‘A 24 h’ is the cell-free area at the 24 h timepoint. Results were presented as percentages relative to the control.

### 2.6. Three-Dimensional Migration Assay

The 3D migration assay was performed using a 6.5 mm Transwell^®^ with 8.0 µm Pore Polyester Membrane Insert (Corning Life Sciences, Corning, NY, USA). In this assay, two medium-filled compartments are separated by a porous membrane through which cells can transmigrate. It allows for the evaluation of the chemotactic responses of cells to a chemoattractant. Initially, 1.1 × 10^5^ A549 cells in 0.5 mL were seeded in 24-well plates and allowed to attach overnight at 37 °C and 5% CO_2_ in the incubator. Then, plates were treated with sham or different irradiation doses of BB (4, 6 Gy) or MRT (EUD to 4 and 6 Gy), and immediately after, cells were harvested and counted. Next, 2.5 × 10^4^ cells were seeded in 0.4 mL of DMEM containing 2% FCS to the insert. DMEM containing 10% FCS (0.75 mL) was added to the external well serving as a chemoattractant. A549 cells were allowed to migrate for 24 h at 37 °C and 5% CO_2_ in the incubator, and then the membrane was fixed with ice cold methanol and stained with crystal violet. Non-migrated cells remaining on the topside of the insert were removed with a cotton swab. Images of at least 5 independent fields (40× magnification) were taken with the BZ-9000 microscope (Keyence, Frankfurt am Main, Germany). The percentage of 3D migrated cells was presented as a percentage of the control, calculated as follows:

(2) $$3D\;migrated\;cells\;\left(\%\right)=\left(\frac{mean\;number\;of\;treated\;cells\;that\;crossed\;the\;membrane\;}{mean\;number\;of\;control\;cells\;that\;crossed\;the\;membrane\;}\right)\times \;100$$

### 2.7. Medium Transfer Experiments

MRC-5 cells (1.5 × 10^5^) in 0.5 mL of 2% FCS medium were seeded in 24-well plates and allowed to attach overnight at 37 °C and 5% CO_2_ in the incubator. Before irradiation, medium was exchanged with 0.5 mL of 10% FCS, and cells were treated with sham or different irradiation doses of BB (4, 6 Gy) or MRT (EUD to 4 and 6 Gy). Cells were incubated for 72 h, and then CM was collected and centrifuged at 15,000 RPM to eliminate any possible detached cells. MRC-5 CM was added at a ratio of 1:3 to A549 cells seeded 24 h before in either 8-well ibidi slides (1.5 × 10^4^ cells in 200 µL/well) for immunofluorescence staining or in 24-well plates (6 × 10^4^ cells in 500 µL/well) for cell collection for quantitative real-time PCR (RTqPCR). For both assays, A549 cells were kept in CM for 72 h.

### 2.8. Co-Culture Assays

Indirect co-culture of MRC-5 and A549 cell lines was performed using Transwells. For migration assays, a 6.5 mm Transwell^®^ with 8.0 µm Pore Polyester Membrane Inserts (Corning, USA) was used. For invasion, 6.4 mm BioCoat^®^ Matrigel^®^ with 8.0 µm PET Membrane Inserts (Corning, USA) was used. For both studies, the method was similar. Initially, 9 × 10^4^ MRC-5 cells in 0.5 mL of a 2% FCS medium were seeded in 24-well plates and allowed to attach overnight at 37 °C and 5% CO_2_ in the incubator. Before irradiation, medium was exchanged for 0.8 mL of 10% FCS medium, and cells were treated with sham or different irradiation doses of BB (4, 6 Gy) or MRT (EUD to 4 and 6 Gy). Cells were incubated for 48 h (for migration) or 24 h (for invasion), and then 2.5 × 10^4^ A549 cells were seeded in 0.4 mL of DMEM containing 2% FCS to the insert. A549 cells were allowed to migrate for 24 h or invade for 48 h at 37 °C and 5% CO_2_ in the incubator. Afterwards, the membrane was fixed with ice cold methanol and stained with crystal violet. Non-migrated/invaded cells remaining on the topside of the membrane were removed with a cotton swab. Images and analyses were performed as reported in Section 2.6.

### 2.9. Quantitative Real-Time PCR (RT-qPCR)

Initially, 6 × 10^4^ A549 or MRC-5 cells in 0.5 mL of 2% FCS medium were seeded in 24-well plates and allowed to attach overnight at 37 °C and 5% CO_2_ in the incubator. Before irradiation, medium was exchanged for 0.5 mL of 10% FCS, and cells were treated with sham or different irradiation doses of BB (4, 6 Gy) or MRT (EUD to 4 and 6 Gy). Irradiated cells were incubated for 72 h and then collected for RTqPCR analysis. For CM experiments, instead of irradiation, A549 cells received the CM as described in Section 2.7. Cells in CM were incubated for 72 h and then collected for RTqPCR. Total RNA was extracted from sham and BB and MRT irradiated cells using the RNeasy^®^ Micro kit from Qiagen (Qiagen, Hilden, Germany), according to the manufacturer’s guidelines. The RNA concentration was measured on a NanoDrop™ Lite Plus spectrophotometer (Thermo Fisher Scientific, Waltham, MA, USA). cDNA synthesis was carried out using the RT^2^ First Strand Kit from Qiagen (Qiagen, Hilden, Germany), according to the manufacturer’s guidelines. Obtained cDNA was subjected to quantitative RT-qPCR on a StepOnePlus device (Applied Biosystems, Foster City, CA, USA) using the QuantiTect^®^ Primmer Assay and RT^2^ SYBR^®^ Green Fluor qPCR Mastermix, both from Qiagen (Qiagen, Hilden, Germany). Primers were directed towards the following migration/invasion-related genes for A549: Transforming growth factor beta (*TGF-β*), nuclear factor kappa-light-chain-enhancer of activated B cells (*NF-κB*), Integrin alpha-V (*ITGAV*), and two Matrix-Metallopeptidases (*MMP2* and *MMP9*). For MRC-5, primers were directed towards *TGF-β* and growth differentiation factor 15 (*GDF-15*). The relative quantification of the genes of interest was computed using the 2^−∆∆CT^ method, normalized against two housekeeping genes, beta-2-microglobulin (*B2M*) and beta-actin (*ACTB*) [24].

### 2.10. Statistical Analyses

Data are presented as the mean ± SD of at least three independent experiments. Statistical analyses were performed by one-way analysis of variance (ANOVA) followed by Tukey’s post-test using GraphPad Prism version 10.6.1 for Windows (GraphPad Software, Boston, MA, USA). The difference was considered statistically significant with a *p* value either less than 0.05, 0.01, or 0.001.

## 3. Results

### 3.1. Irradiation

**Figure 1:**
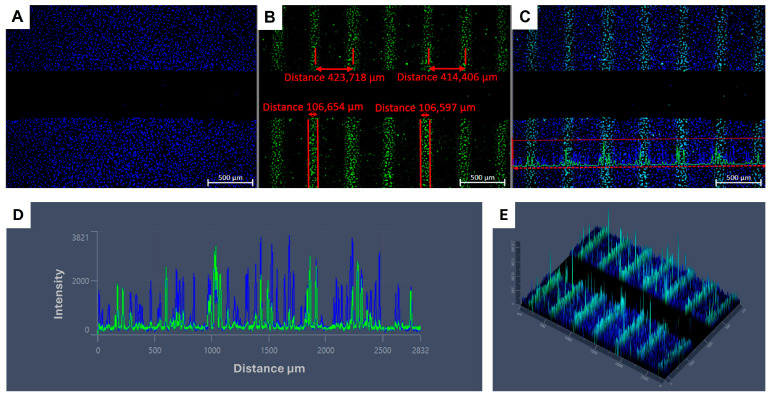
ɣH2Ax staining in A549 cell line 30 min post irradiation (pi) with 6 Gy (EUD) MRT for a 2D migration assay. Stained nuclei (Hoechst, blue) in high confluency around a central cell-free gap (**A**). ɣH2Ax staining (Alexa Fluor^®^ 488, green) showing the microbeam pattern. High-ɣH2ax-intensity (peaks) measuring around 110 µm, with a ctc distance of around 420 µm, are observed perpendicular to cell-free gap (**B**). Merged Hoechst/Alexa Fluor^®^ 488 image with a highlighted linear region of interest (red dashed line) (**C**). Intensity versus distance profile shows the signal distribution along the linear region of interest depicted on ‘C’ (**D**). A 2.5D intensity map illustrating the ɣH2ax distribution across the irradiated sample (**E**).

Immunofluorescent gH2AX staining for DNA damage on confluent A549 cells showed the microbeam configuration. With the irradiation setup used, the beam width was approximately 106 µm, and the ctc distance was around 420 µm on the biological sample, 30 min post MRT irradiation, as shown in Figure 1.

### 3.2. BB and MRT Doses Applied Do Not Affect A549 or MRC-5 Cell Viability Within 72 h

**Figure 2:**
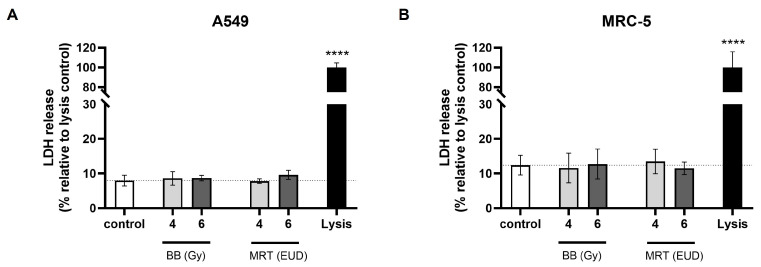
Percentage of LDH release relative to lysis control in A549 (**A**) and MRC-5 (**B**) cells. Cells were irradiated with 4 Gy or 6 Gy of BB and the EUD of MRT. LDH was quantified at 72 h pi. Data are presented as the mean ± SD of at least three separate experiments. **** *p* < 0.0001.

When performing migration/invasion studies, it is important that treatment doses applied have no cytotoxic effect within the study timeframe. Thus, it is possible to distinguish between reductions in motility and cell death [7]. Irradiation doses used in the present study did not increase the LDH release as compared to the spontaneous release levels for non-irradiated cells, as shown in Figure 2. This shows the non-cytotoxicity of the BB and MRT doses applied within a 72 h timeframe for both A549 and MRC-5 cell lines.

### 3.3. Irradiation with BB and MRT Increases A549 Cell Size and Adhesion

**Figure 3:**
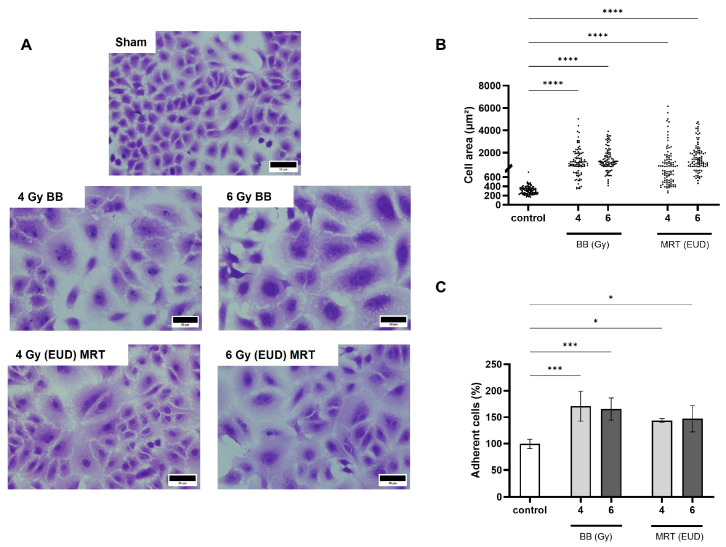
BB and MRT irradiation induces changes in the morphology and adhesion of A549 cells. Differences in cell morphology of crystal violet stained cells observed in bright field, 72 h pi, 40×, scale bar = 50 µm (**A**). Differences in cell area at 72 h pi (**B**). Percentage of adherent A549 cells relative to non-irradiated control, 30 min pi (**C**). Data are presented as the mean ± SD of at least three separate experiments. * *p* < 0.05, *** *p* < 0.001 and **** *p* < 0.0001.

In the present study, a remarkable change in cell morphology 72 h post irradiation (pi) was observed for both BB and MRT (Figure 3A). Non-irradiated A549 cells presented the typical cobblestone morphology, with an average cell size of approximately 300 µm^2^. This area was increased to an average of approximately 1300 µm^2^ and 1500 µm^2^ 72 h pi with 4 Gy and 6 Gy BB, respectively (*p* < 0.0001). Cells irradiated with MRT EUD to 4 Gy and 6 Gy showed similar dose-dependent cell area increases to averages of approximately 1250 µm^2^ and 1650 µm^2^, respectively (*p* < 0.0001; Figure 3B). We also observed a significant increase in A549 adhesion as compared to the non-irradiated control, regardless of the dose and irradiation modality applied. Irradiation with 4 Gy and 6 Gy BB led to average increases of around 70% and 65% in adhesion, respectively, as compared to the control (*p* < 0.001). The MRT EUD of 4 Gy and 6 Gy increased adhesion with averages of around 44% and 48%, respectively (*p* < 0.05; (Figure 3C).

### 3.4. A549 Irradiation with BB and MRT Affects Cell Migration in a Distinct Manner

**Figure 4:**
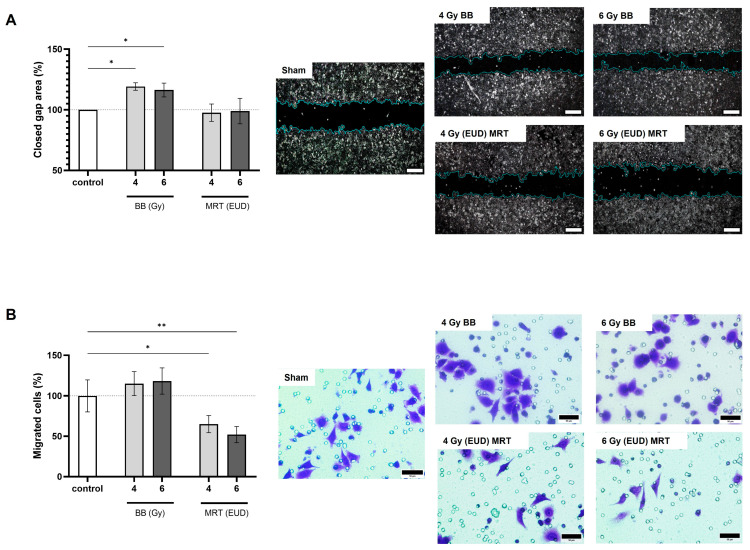
Differential motility response to BB and MRT irradiation. BB but not MRT increases 2D migration of A549 cells, scale bar = 300 µm (**A**). Only MRT leads to significant reduction in 3D migration of A549 cells, scale bar = 50 µm (**B**). Cells were allowed to migrate for 24 h after irradiation in both studies. Data are presented as the mean ± SD of at least three separate experiments. * *p* < 0.05 and ** *p* < 0.01.

On the 2D assay (Figure 4A), we observed that only BB irradiation led to a significant increase in cell migration. The percentage of 2D migrated cells was on average 19% and 16% higher after BB 4 Gy and BB 6 Gy (*p* < 0.05), respectively, as compared to the control. Two-dimensional migration was not affected by MRT irradiation (*p* > 0.05). On the 3D assay (Figure 4B), a non-significant increase in migration was observed after BB irradiation (*p* > 0.05). On the other hand, MRT irradiation led to a significant decrease in cells that were able to cross the porous membrane. The percentage of 3D migrated cells after MRT EUD 4 Gy and 6 Gy was on average 35% (*p* < 0.05) and 48% (*p* < 0.01) lower, respectively, as compared to the control.

### 3.5. MRT Irradiation of A549 Cells Downregulates NF-κB and Prevents Increase in CD44

**Figure 5:**
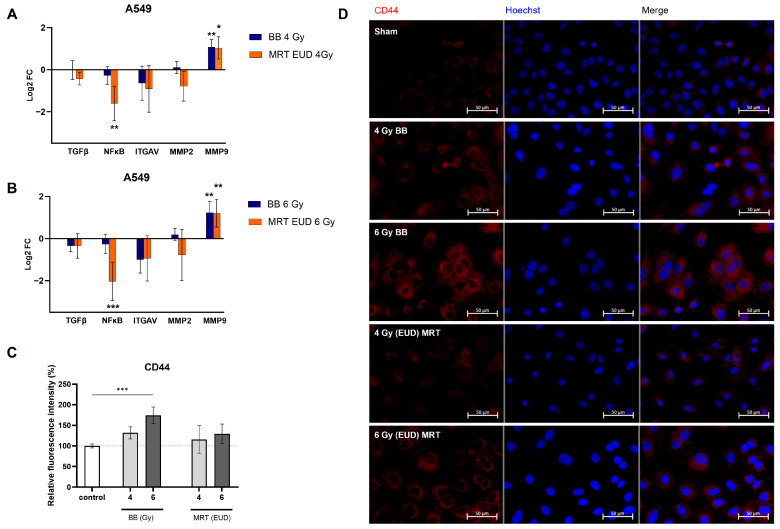
Impact of BB and MRT irradiation on the expression of different migration-related genes and CD44 protein in A549 cells. Logarithm of fold change (Log2FC) for five differentially expressed migration-related genes in A549 cells after irradiation with 4 Gy (**A**) or 6 Gy. (**B**) of either BB or MRT EUD. Relative fluorescence intensity of CD44 quantified in A549 cells (**C**). Representative fluorescence images of CD44 expression in A549 cells. Red: Alexa Fluor 647, CD44; blue: Hoechst, nuclei, scale bar = 50 µm (**D**). A549 cells were exposed to either BB or MRT at doses of 4 Gy and 6 Gy EUD and evaluated 72 h post exposure. Data are presented as the mean ± SD of at least three separate experiments. * *p* < 0.05; ** *p* < 0.01; *** *p* < 0.001.

The expression of *TGF-β*, *ITGAV*, and *MMP2* was not significantly affected by irradiation with BB or MRT. Both BB and MRT led to a similar increase in *MMP9* expression (Log2FC > 1.0) for both doses evaluated (*p* < 0.05). *NF-κB* expression was differently modulated by BB and MRT. While no difference in *NF-κB* levels was observed for BB-irradiated A549 as compared to the control (*p* > 0.05), a significant downregulation was observed for treatments with 4 Gy EUD MRT (Log2FC < −1, *p* < 0.01) and 6 Gy EUD MRT (Log2FC < −2, *p* < 0.001). RTqPCR results are presented in Figure 5A,B. CD44 expression was also differently modulated by BB and MRT. A significant increase in CD44 expression (Figure 5C,D) occurred only when A549 cells received a dose of 6 Gy BB (*p* < 0.001). Irradiation with 4 Gy BB and either dose of MRT (EUD to 4 and 6 Gy) did not result in a significant CD44 increase (*p* > 0.05).

### 3.6. Conditioned Medium from MRC-5 Influences Motility Pathways in A549 Cells

It is known that the tumor microenvironment (TME) plays an important role in modulating hallmarks of cancer progression, including metastasis [25]. Herein, we investigated the impact of exposing A549 cells to CM of MRC-5 irradiated with different RT modalities.

**Figure 6:**
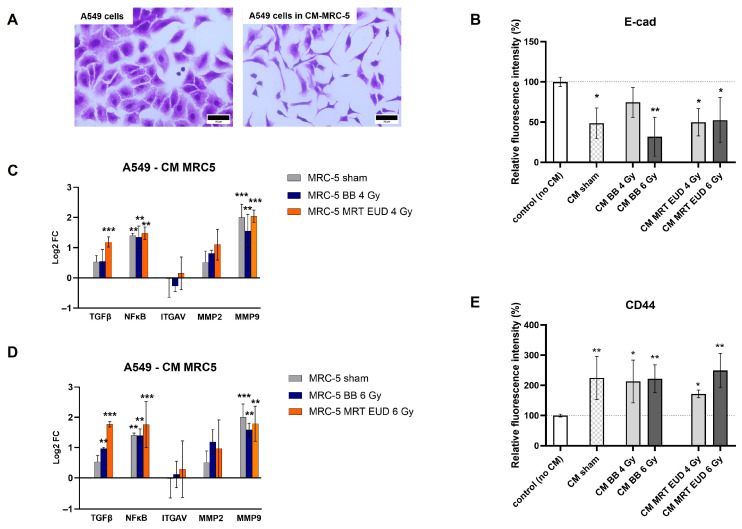
Impact of MRC-5 CM on A549 cells. Morphological changes observed in A549 cells (**A**). Relative fluorescence intensity of E-cad in A549 cells exposed to different MRC-5 CM (**B**). Logarithm of fold change (Log2FC) for five differentially expressed migration-related genes in A549 cells exposed to MRC-5 CM after irradiation with 4 Gy (**C**) or 6 Gy. (**D**) of either BB or MRT EUD. Relative fluorescence intensity of CD44 in A549 cells exposed to different MRC-5 CM (**E**). A549 cells were exposed to CM for 72 h. MRC-5 cells were either sham, irradiated with BB doses of 4 Gy and 6 Gy, or the EUD of MRT. CM was collected 72 h post MRC-5 irradiation. Data are presented as the mean ± SD of at least three separate experiments. * *p* < 0.05; ** *p* < 0.01; *** *p* < 0.001. CM: conditioned medium. Scale bar = 50 µm.

Figure 6A presents the change in A549 morphology when exposed to MRC-5 sham conditioned medium. In the presence of MRC-5 CM, independent of treatment, A549 cells lost their typical cobblestone morphology, becoming elongated, with a fibroblast-like morphology. We also verified that the expression of the epithelial marker E-cadherin decreased significantly (*p* < 0.05) for all groups except CM BB 4 Gy (*p* > 0.05; Figure 6B, Supplementary Figure S1).

The regulation of migration/invasion-related genes upon exposure of A549 to different MRC-5 CM is presented on Figure 6C,D. The expression levels of *ITGAV* and *MMP2* on A549 cells were not significantly affected by their exposure to CM of MRC-5 cells either non-irradiated or irradiated with different doses of BB or MRT (*p* > 0.05). An upregulation of *NF-κB* and *MMP9* (*p* < 0.01) occurred for all groups, including MRC-5 CM sham, proving to be independent of irradiation. The *TGF-β* expression, however, was upregulated significantly only when A549 cells were exposed to CM of irradiated MRC-5 with either 6 Gy BB (*p* < 0.01) or to both doses of MRT (*p* < 0.001). The TGF-*β* upregulation observed after MRT in EUD to 4 Gy and 6 Gy was significantly higher than that observed for the same doses of BB (*p* < 0.05).

The CD44 expression was also not dependent on previous irradiation of MRC-5. An average increase of 125% was observed for MRC-5 CM sham (*p* < 0.05; Figure 6E, Supplementary Figure S2), with no significant difference from the other groups.

### 3.7. Co-Culture with Irradiated MRC-5 Does Not Significantly Impact A549 3D Migration

Cancer-associated fibroblasts (CAFs) are the major component of the TME, accounting for almost 70% of the cells in the tumor tissue [26]. To investigate the interactions between MRC-5 and A549 and how they impact the migration of the latter, we performed an indirect co-culture assay using Transwells.

**Figure 7:**
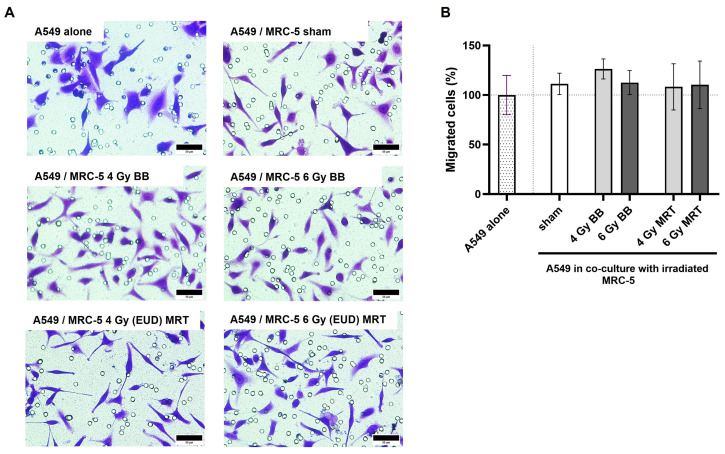
Indirect co-culture with MRC-5 cells does not impact significantly the 3D migration of A549. Co-culture with MRC-5 leads to changes in morphology of A549 cells (**A**). Three-dimensional migration of A549 cells is not impacted by co-culture with MRC-5, independently of its irradiation status (**B**). Cells were allowed to migrate for 24 h after co-culture was established. MRC-5 cells were either sham, irradiated with BB doses of 4 Gy and 6 Gy, or the EUD of MRT. Data are presented as the mean ± SD of at least three separate experiments. Scale bar = 50 µm.

When in co-culture, the same morphology change observed for A549 cells exposed to MRC-5 CM was seen for the cells crossing the membrane. As shown in Figure 7A, cells in co-culture presented a more elongated, fibroblast-like morphology as compared to A549 alone with its typical cobblestone morphology. However, no significant difference in migration was observed for A549 cells in co-culture with MRC-5, independently of the irradiation status of the MRC-5 cell line (*p* > 0.05; Figure 7B).

### 3.8. Co-Culture with MRT Irradiated MRC-5 Leads to Increased A549 Invasion

**Figure 8:**
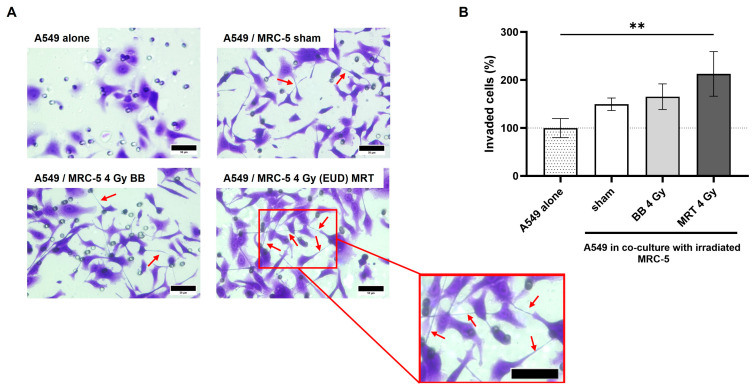
Indirect co-culture with MRC-5 cells impacts the invasion of A549. Changes in morphology of A549 cells are observed on co-culture invasion assay, with many cytoplasmic extensions that resemble tunneling nanotubes (**A**). A549 invasion was significantly increased when in co-culture with MRT irradiated MRC-5 (**B**). Cells were allowed to invade for 48 h after co-culture was established. MRC-5 cells were either sham, irradiated with BB dose of 4 Gy, or the EUD of MRT. Data are presented as the mean ± SD of at least three separate experiments. ** *p* < 0.01. Red arrows indicate cytoplasmic extensions resembling tunneling nanotubes. Scale bar = 50 µm.

In the invasion study, cytoplasmic extensions connecting proximal or distant cells were visualized in great numbers for A549 cells in co-culture (Figure 8A). An increase in invaded cells was observed for all groups in co-culture, as compared to A549 alone (Figure 8B). Although groups in co-culture do not differ statistically among themselves (*p* > 0.05), when compared to the A549 alone, the only significant increase occurred to A549 in co-culture with MRC-5 cells irradiated with 4 Gy (EUD) MRT (*p* < 0.01).

### 3.9. Fibroblast Irradiation

**Figure 9:**
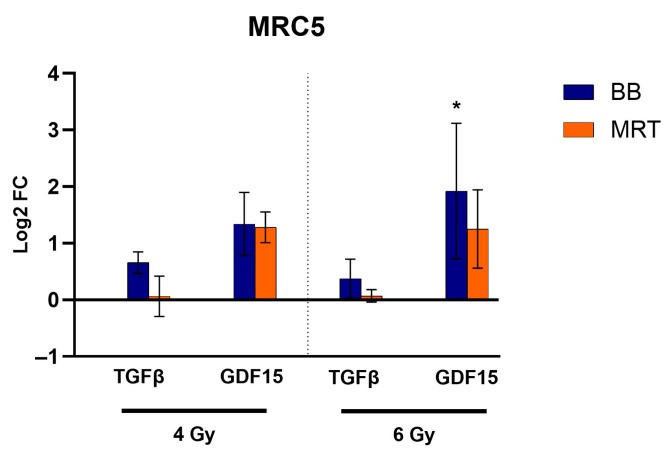
Impact of BB and MRT irradiation on MRC-5 cells. Logarithm of fold change (Log2FC) for two differentially expressed genes in MRC-5 cells exposed to either BB doses of 4 Gy and 6 Gy or the EUD of MRT. Data are presented as the mean ± SD of at least three separate experiments. * *p* < 0.05.

Expression levels of *TGF-β* on MRC-5 post irradiation with either BB or MRT remained unchanged. A trend towards upregulation of *GDF15* was observed for both BB and MRT irradiation; however, it was only significant for cells irradiated with 6 Gy BB (*p* < 0.05). RTqPCR results for *TGF-β* and *GDF-15* are presented in Figure 9.

## 4. Discussion

While having a central role in cancer management, the potential influence of radiotherapy as a driver of cancer metastasis remains a subject of controversy [27]. There is evidence that ineffective RT could enhance the metastatic potential of solid tumors due to phenotypical and genetical modification of the irradiated cells [6].

In the present study, A549 and MRC-5 cell lines were treated with BB and MRT irradiation in doses that proved to be non-toxic in the timeframe (72 h) of the studies. Irradiated A549 cells went through a remarkable change in cell morphology for both BB and MRT irradiation, with their size increasing significantly. Cell spreading, with an increase in adhesive area, is one of the indirect indicators of cell adhesion strength [28]. Enhanced adhesion properties enable tumor cells to bind more effectively to the extracellular matrix (ECM), fostering their invasive potential. This interplay between tumor cells and the ECM is critical for activating signaling pathways responsible for regulating cell motility, preconditioning them for a subsequent metastasis cascade [29]. Ionizing radiation is known to alter the cell adhesion of different tumor entities [30]. The morphological transition of cells from spherical to flat due to structural cytoskeletal modifications is the main observable change during cell adhesion [31]. We indeed observed a significant increase in A549 adhesion as compared to the non-irradiated control, not dependent on the dose and irradiation modality applied. These results are in accordance with those demonstrated by Jung et al. (2007), in whose study BB irradiation also led to an increase in A549 cell adhesion [8].

Cell migration is one of the first steps in the metastatic cascade, and metastasis is a major cause of LC treatment failure [32,33]. Therefore, we investigated the impact of BB and MRT on the 2D and 3D migration abilities of the A549 cells. In a 2D assay, cells are only required to move horizontally, and there is no nutrient gradient present. Meanwhile, on the 3D assay, they need not only to move around to find a pore but also to squeeze through it as part of a chemotactic response toward a chemoattractant (in this case, a higher concentration of FCS). The interplay between adhesion and contractility required in these assays influences the types of protrusion that the cells use to migrate, modulated by different biological pathways [34]. We observed an increase in A549 2D migration post BB irradiation with both doses. BB irradiation has previously been demonstrated to increase A549 2D migration [8,9,10,11,12]. This stimulation was not observed when either dose of MRT was applied. Although BB irradiation did not significantly increase 3D migration, MRT irradiation significantly reduced the number of cells that were able to cross the membrane. The only study we found reporting the effect of BB irradiation on the 3D migration of A549 cells was the one by Li and coworkers (2000), reporting a significant increase in migration after irradiation with 4 Gy BB [10]. Taken together, these results indicate that MRT is effective in limiting or preventing cellular migration.

In the present study, *TGF-β*, *ITGAV*, and *MMP2* did not have their expression altered when A549 cells were irradiated by BB or MRT. *MMP9* expression was increased in a similar manner for both BB and MRT. *MMP9* is a member of the zinc metalloproteinase family responsible for catalyzing the degradation of elastin and collagens from the ECM, thereby fostering migration [35]. A study by Zhao and coworkers (2017) also reported *MMP9* upregulation on A549 cells after 4 Gy BB irradiation [12]. BB and MRT irradiation differently modulated *NF-κB* expression in A549 cells. *NF-κB* plays a critical role for cancer progression, enhancing the expression of adhesion molecules and degradative enzymes contributing to metastasis. In patients with non-small-cell lung cancer (NSCLC), *NF-κB* is considered an unfavorable prognosis and survival marker. DNA double-strand breaks are known to activate the ataxia telangiectasia mutated (ATM) pathway, leading to *NF-κB* activation [36,37]. *NF-κB* activation by irradiation is described as a dose-dependent event, which usually requires doses above 7 Gy [38]. Under the conditions and radiation doses applied in this study, *NF-κB* upregulation was not observed. In the present study, no difference in *NF-κB* levels was observed for BB-irradiated samples as compared to the control. More intriguing was the significant downregulation of *NF-κB* observed for treatments with MRT. Different negative feedback mechanisms induced by DNA damage limiting the genotoxic *NF-κB* activation have been reported by Wang and coworkers [39]. Further studies investigating which subset of *NF-κB* genes is activated after irradiation with each of the different modalities would be necessary to confirm if negative feedback is responsible for the MRT downregulation of *NF-κB* in A549 cells observed herein. The genetic background of the A549 cell line can also play an important role in these findings. These cells are p53 wildtype and carry a KRAS mutation [38]. The *NF-κB* pathway plays a role in promoting EMT, while p53 has been demonstrated to suppress it. Mutant p53 has been associated with increasing activity of *NF-κB* by prolonged stabilization on κB sites; therefore, p53 mutated cells might respond differently [40]. Meanwhile, activation of *NF-κB* pathways is associated with mutant *KRAS*, indicating that *KRAS* wild-type cell lines might not benefit to the same extent as seen herein [41,42]. In a slightly different context, using α-particle microbeam irradiation, Hellweg and coworkers (2007) reported the activation of *IκBα* expression on human embryonic kidney cells (HEK/293). This protein is known to be responsible for the termination of radiation-induced *NF-κB* activation, one of the possible negative feedback mechanisms [43]. The only report on the modulation of *NF-κB* by photon MRT available refers to a melanoma in vivo model, where MRT led to significantly overexpression of *NF-κB* [44]. This could indicate that the responses are radiation/cell line/entity specific. It is also important to highlight that the single timepoint of 72 h was evaluated in the present study. Studies of additional timepoints will allow the characterization of the *NF-κB* modulation dynamics immediately post-MRT. CD44 is a non-kinase cell surface transmembrane glycoprotein, a member of the cell adhesion molecule family. It plays a role in cell growth and proliferation, epithelial–mesenchymal transition (EMT), adhesion, migration, and invasion [45]. Overexpression of CD44 in NSCLC in vitro has been associated with cell proliferation, migration, and invasion [46,47,48]. A recent meta-analysis showed that CD44 is an effective prognostic factor for NSCLC. Although its overexpression has been linked to tumor differentiation and a worse patient survival rate, no relationship was found between CD44 and NSCLC metastasis [49]. Gomez-Casal (2013) demonstrated that spheroids of NSCLC cells (H460 and A549) that survive irradiation treatment (5 Gy BB) present a strong upregulation of CD44, suggesting it can be used as a predictive marker for recurrence after radiotherapy [50]. In the present study, we observed a significant increase in CD44 expression only when A549 cells received a dose of 6 Gy BB. Irradiation with 4 Gy BB and EUD to 4 Gy and 6 Gy MRT did not result in a significant CD44 increase. CD44 has been demonstrated to be a downstream target of *NF-κB* [51]. In a study by Smith and coworkers (2014), a direct correlation with *NF-κB* inhibition and CD44 repression was established, resulting in decreased invasiveness of breast cancer cells. However, the molecular mechanism behind CD44 regulation by *NF-κB* remains controversial [52]. MRC-5-CM has previously been reported to promote cell motility and the invasiveness of different tumor entities [53,54]. In the present study, the change in morphology to an elongated, fibroblast-like shape as well as loss of E-cadherin are features observed for A549 undergoing EMT [22,55,56]. We observed an increase in *NF-κB* and *MMP9* in A549 cells exposed to MRC-5 CM, which was independent of irradiation of the latter. CD44 also increased for all groups once, again indicating a correlation with *NF-κB* levels. An increase in *TGF-β* transcription was observed after exposure to irradiated MRC-5 CM. This indicates that irradiation of fibroblasts leads to EMT program activation in A549 cells, which can afterwards be regulated by autocrine signaling. This contributes to maintaining the mesenchymal state and motility [57]. This consists of a deleterious effect of irradiating the TME, which, in the present study, was observed more prominently after MRT irradiation. Indirectly co-culturing A549 cells with MRC-5 cells led to changes in A549 morphology similar to those observed when it was exposed to MRC-5 CM. Although no change in A549 3D migration was observed, invasion was increased when in co-culture. This increase was only significant when MRC-5 were exposed to MRT irradiation, once again indicating a detrimental effect of MRT irradiation of the TME. The cytoplasmic extensions connecting A549 invaded cells in the co-culture study resemble tunneling nanotubes (TNTs), thin membranous tubes that interconnect cells, allowing direct communication. Structures such as TNTs and tumor microtubes are stimulated by EMT and play an important role in cancer progression [58]. TNTs have previously been reported in A549 as well as in primary lung adenocarcinoma cells derived from a human patient sample [59,60,61,62]. In the present study, these TNT-like structures were more developed in the invasion co-culture assay in comparison with the migration co-culture assay, possibly due to the higher complexity of the matrix on the former. This dependency on the substrate for the formation and type of cellular protrusions has previously been described by Franchi and coworkers (2020) [63]. Additionally, it is known that in radiation-induced fibrosis, fibroblasts are activated into myofibroblasts, a process that is triggered mainly by transforming growth factors (TGFs) [64]. Therefore, we investigated the impact of BB and MRT irradiation on the expression levels of *TGF-β* and *GDF15* (members of the *TGF-β* family) in the MRC-5 cell line. *TGF-β* is behind many interactions within tumor and normal tissue. It plays a role in the differentiation of fibroblasts into cancer-associated fibroblasts as well as in tumor cell invasion by activating EMT. *TGF-β* upregulation in fibroblasts plays an important role in radiation-induced fibrosis [65,66]. Herein, irradiation of MRC-5 with either BB or MRT did not lead to changes in the expression levels of *TGF-β* within 72 h. As *TGF-β* is a classic EMT driver, this might explain why the EMT features observed in A549 cells when in CM of MRC-5 (fibroblast-like shape and loss of E-cadherin) were independent of MRC-5 irradiation. However, it is important to keep in mind that immediate changes in the protein levels in the medium might take place independently of post-transcriptional regulation. Assays such as ELISA will be necessary to quantify the levels of *TGF-β* secreted in the CM. GDF15 has been reported to regulate fibroblast function, participating in lung fibrosis [67]. In a study by Herskind and coworkers (2021), *GDF15* was strongly upregulated in human skin fibroblasts 72 h post irradiation with 4 Gy BB [68]. In our study, *GDF15* was only significantly upregulated when MRC-5 received BB radiation at a dose of 6 Gy.

In summary, we confirmed the detrimental effect of BB irradiation to cell motility and showed that MRT affects cell migration in a distinct manner, possibly related to the differential modulation of *NF-κB*/CD44 expression. MRT proved to be a promising strategy when only the direct irradiation of the A549 cells is taken into account. On the other hand, MRT irradiation of MRC-5 lung fibroblasts indirectly impacts A549 motility, with more detrimental consequences compared to BB irradiation. At present, the radiobiological mechanisms behind the favorable antitumor response of MRT are not fully understood [69]. In particular, elucidating their differential modulation of cell motility will require dedicated radiobiological studies.

Our study is the first of its kind, giving important insights into MRT research. However, it presents some limitations. The results obtained with the A549 cells might be representative of the behavior observed for cells with a similar genetic background, but not all NSCLCs. In the future, it is important to investigate a panel of cell lines with different genetic statuses. This will allow us to verify if the radiation effects are gene expression status-dependent [70]. The timepoints post irradiation also play an important role on the outcomes of motility. Motility changes in response to irradiation can be reversed or intensified in a few days. Herein, the effects were verified up to 72 h. Ultimately, the systems we used do not fully recapitulate the complexity and heterogeneity of the TMEs, with different cell populations, compositions, and architectures, which play a role in cell behavior [30]. For other tumor entities, increased cell motility observed in vitro after BB irradiation has been widely demonstrated to be recapitulated in vivo [7]. In the future, more sophisticated models will allow for a better comparison of the effects of BB and MRT on different tumor tissues.

## 5. Conclusions

This study was the first to investigate the direct and indirect impacts of MRT irradiation on the motility of a tumor cell line. A number of previous studies have demonstrated the detrimental effects of BB irradiation on A549 cell motility, as confirmed in the present study. In this context, MRT proves to be a promising strategy, as direct irradiation of A549 cells with MRT limited or prevented cellular migration. However, the overall scenario appears to be significantly more complex. Irradiation of MRC-5 cells with MRT and its interactions with the A549 cell line led to a TGF-β upregulation on the tumor cell line, which was significantly higher compared to BB. It also led to a significant increase in the invasion of co-cultured A549 as compared to the control. The findings of this study highlight the importance of the investigation of the effects of MRT irradiation not only on the tumor but also its microenvironment. Further validation across a panel of NSCLC cell lines with distinct genetic backgrounds is necessary to confirm the reported effects. In vivo models of NSCLC focusing on metastasis after irradiation will allow us to fully understand how BB and MRT impact NSCLC progression.

## Data Availability

The original contributions presented in this study are included in the article/Supplementary Material. Further inquiries can be directed to the corresponding authors.

## Supplementary Materials

The following supporting information can be downloaded at https://www.mdpi.com/article/10.3390/cells15020107/s1, Figure S1: Impact of MRC-5 CM on the E-cadherin expression in A549 cells; Figure S2: Impact of MRC-5 CM on the CD44 expression in A549 cells.

## Abbreviations

The following abbreviations are used in this manuscript:

ACTB	Beta-actin
ANOVA	One-way analysis of variance
B2M	Beta-2-microglobulin
BB	Broad beam
CAFs	Cancer-associated fibroblasts
CM	Conditioned medium
Ctc	Center-to-center
DMEM	Dulbecco’s modified Eagle medium
ECM	Extracellular matrix
EMT	Epithelial–mesenchymal transition
EUD	Equivalent uniform dose
FCS	Fetal calf serum
GDF-15	Growth differentiation factor 15
ITGAV	Integrin alpha-V
LC	Lung cancer
Log2FC	Logarithm of fold change
MMP2	Matrix-Metallopeptidase 2
MMP9	Matrix-Metallopeptidases 9
MRT	Microbeam radiation therapy
NF-κB	nuclear factor kappa-light-chain-enhancer of activated B cells
NSCLC	Non-small-cell lung cancer
PBS	Phosphate-buffered saline
Pi	Post-irradiation
PVDR	Peak-to-valley dose ratio
RT	Radiotherapy
RTqPCR	Quantitative real-time PCR
TGF-β	Transforming growth factor beta
TME	Tumor microenvironment
TNT	Tunneling nanotube
