# Supplementary material for: Targeting Lung Cancer Cell Motility Using Microbeam Radiation Therapy
Source: Cells. 2026 Jan 7;15(2):107. doi: 10.3390/cells15020107 (PMC12839417; doi:10.3390/cells15020107)
Supplement: Supplementary file 1 [file cells-15-00107-s001.zip › cells-4069752-supplementary.pdf]

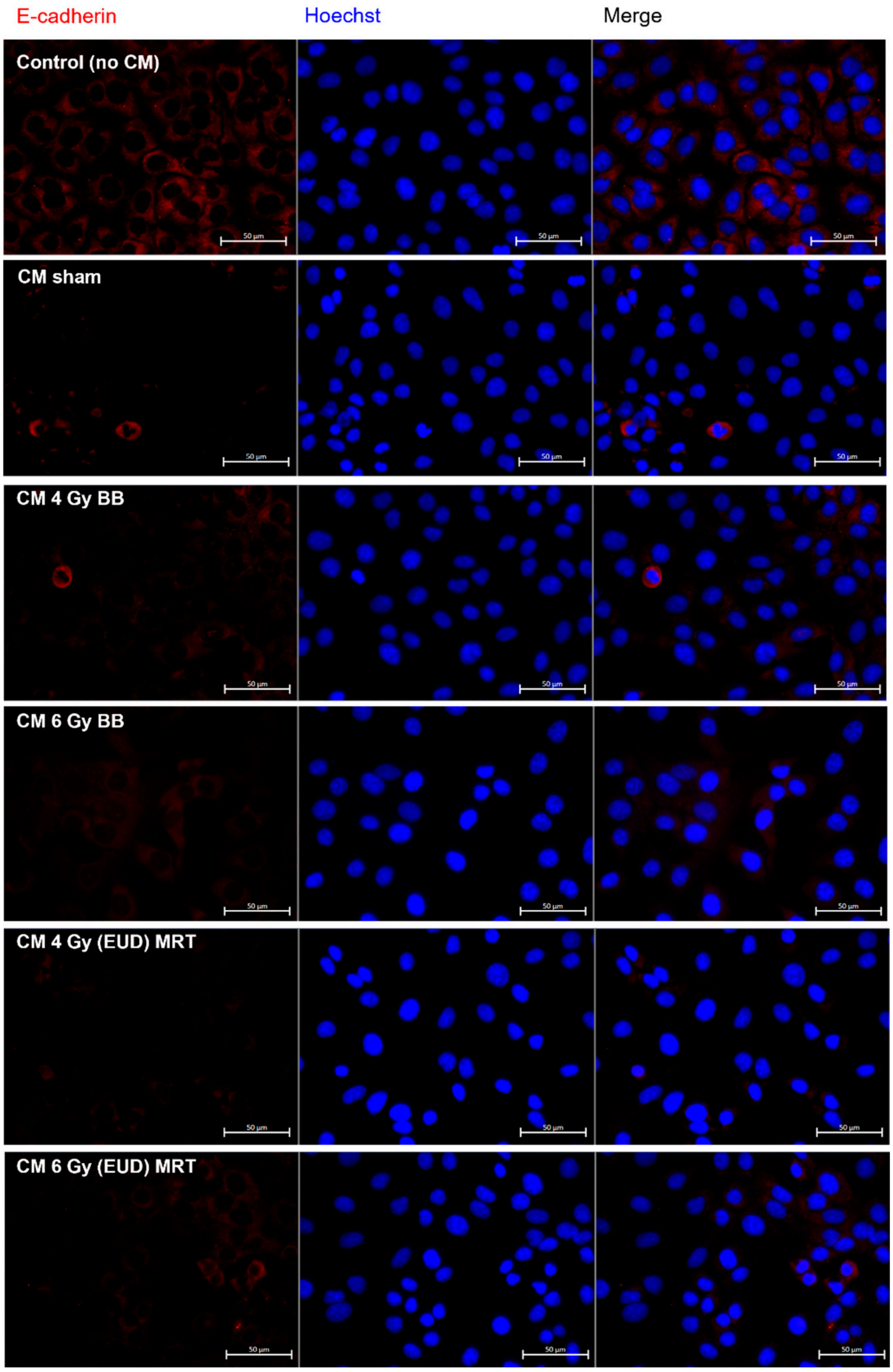

Supplementary Figure S1: Impact of MRC-5 CM on the E-cadherin expression in A549 cells. Representative fluorescence images of E-cadherin expression in A549 cells after exposure to MRC-5 CM for 72 hours. MRC-5 cells were either sham, irradiated with BB doses of 4 Gy and 6 Gy or the EUD of MRT. CM was collected 72 hours post MRC-5 irradiation. Red: Alexa Fluor 647, E-cadherin; blue: Hoechst, nuclei.

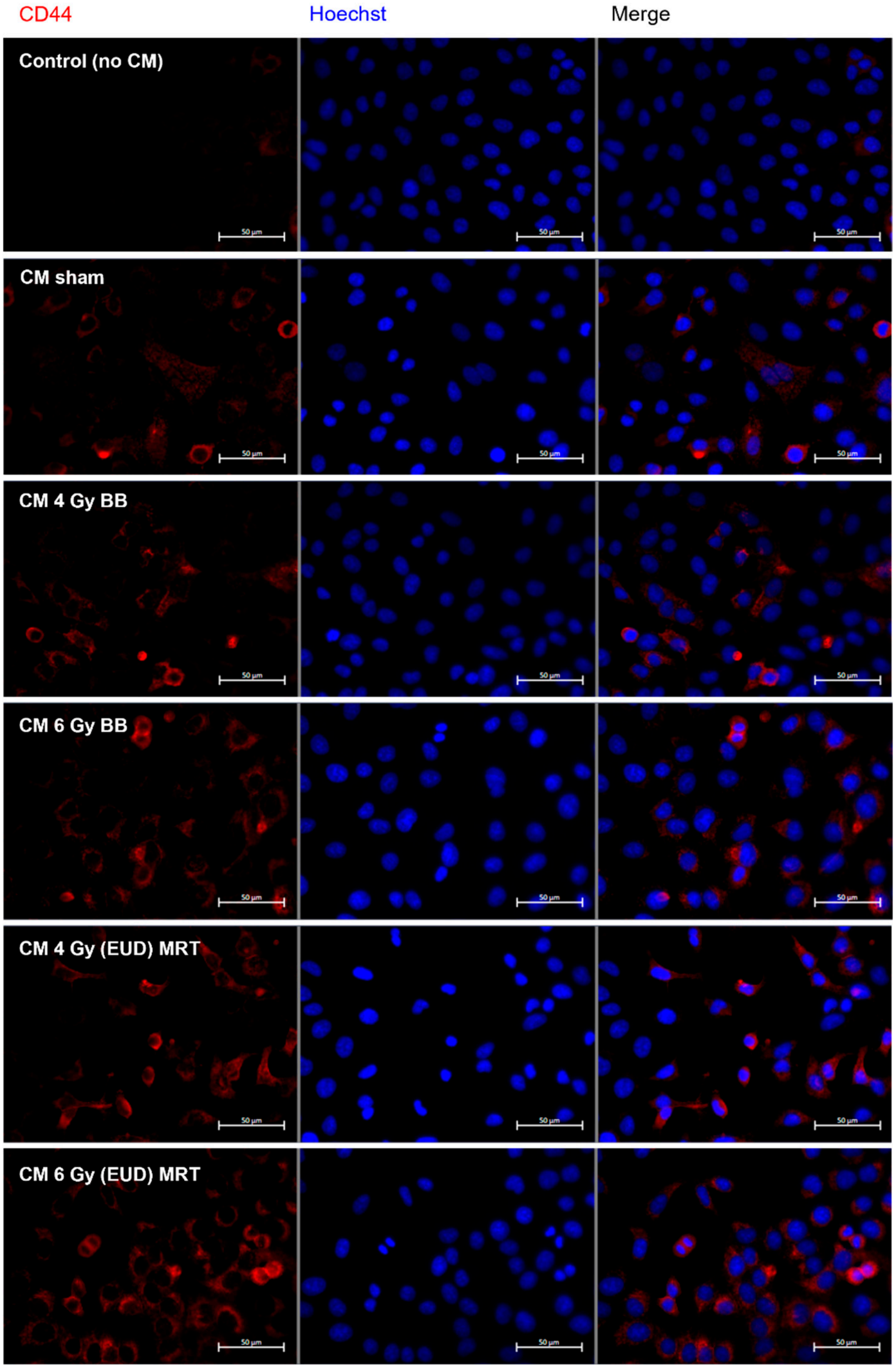

Supplementary Figure S2: Impact of MRC-5 CM on the CD44 expression in A549 cells. Representative fluorescence images of CD44 expression in A549 cells after exposure to MRC-5 CM for 72 hours. MRC-5 cells were either sham, irradiated with BB doses of 4 Gy and 6 Gy or the EUD of MRT. CM was collected 72 hours post MRC-5 irradiation. Red: Alexa Fluor 647, CD44; blue: Hoechst, nuclei.
